# Author Correction: Analogous response of temperate terrestrial exoplanets and Earth’s climate dynamics to greenhouse gas supplement

**DOI:** 10.1038/s41598-023-42703-z

**Published:** 2023-09-18

**Authors:** Assaf Hochman, Thaddeus D. Komacek, Paolo De Luca

**Affiliations:** 1 Fredy and Nadine Hermann Institute of Earth Sciences, The Hebrew University of Jerusalem, Jerusalem, Israel; 2 Department of Astronomy, The University of Maryland, College Park, USA; 3 Barcelona Supercomputing Center, Barcelona, Spain

Correction to: *Scientific Reports* https://doi.org/10.1038/s41598-023-38026-8, published online 10 July 2023

The original version of this Article contained an error in Figure 5 (i) x-axis label.

“WS”

now reads:

“T”

The original Figure 5 and accompanying legend appear below.

**Figure 5:**
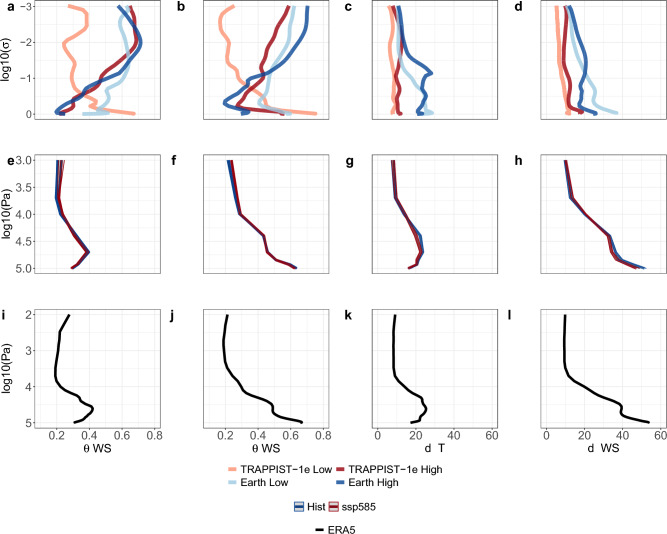
Global dynamical systems metrics vertical profiles for temperature (T) and wind speed (WS). (**a**–**d**) TRAPPIST-1e and Earth-like ExoCAM simulation averages under high and low pCO_2_ scenarios. (**e**–**h**) CMIP6 multi-model ensemble medians for the historical (1981–2010) and SSP5-8.5 (2071–2100) periods. (**i**–**l**) Average of ERA5 reanalysis for the historical period (1981–2010). Dynamical systems metrics are local inverse persistence (*θ*) and local dimension (*d*; see Methods).

The original Article has been corrected.

