# Supplementary material for: Analogous response of temperate terrestrial exoplanets and Earth’s climate dynamics to greenhouse gas supplement
Source: Sci Rep. 2023 Jul 10;13:11123. doi: 10.1038/s41598-023-38026-8 (PMC10333385; doi:10.1038/s41598-023-38026-8)
Supplement: Supplementary file 1 — Supplementary Information. [file 41598_2023_38026_MOESM1_ESM.pdf]

Scientific Reports

Supplementary material for:

Analogous response of temperate terrestrial exoplanets and Earth's climate dynamics to greenhouse gas supplement

Assaf Hochman[1*], Thaddeus D. Komacek[2], Paolo De Luca[3]

*Corresponding author. Email: assaf.hochman@mail.huji.ac.il

1. Fredy and Nadine Hermann Institute of Earth Sciences, The Hebrew University of Jerusalem, Jerusalem, Israel.
2. The University of Maryland, Department of Astronomy, College Park, USA.
3. Barcelona Supercomputing Center, Barcelona, Spain.

Contents of this file:

Table S1 and Figure S1

Table S1 List of Coupled Model Intercomparison Project phase-6 (CMIP6) models used in the analyses for both historical (1981-2010) and future (SSP5-8.5; 2071-2100) simulations. The table includes the following columns: Institution, model, nominal resolution, member, reference, and DOI.

**Institution**	**Model**	**Nominal resolution**	**Member**	**Reference**	**DOI**
CSIRO-ARCCSS	ACCESS-CM2	250km	r1i1p1f1	Bi et al. (2020)	https://doi.org/10.1071/ES19040
CSIRO	ACCESS-ESM1-5	250km	r1i1p1f1	Ziehn et al. (2020)	https://doi.org/10.1071/ES19035
CMCC	CMCC-CM2-SR5	100km	r1i1p1f1	Cherchi et al. (2019)	https://doi.org/10.1029/2018MS001369
CMCC	CMCC-ESM2	100km	r1i1p1f1	Lovato et al. (2022)	https://doi.org/10.1029/2021MS002814
MIROC	MIROC6	250km	r1i1p1f1	Tatebe et al. (2019)	https://doi.org/10.5194/gmd-12-2727-2019
MPI-M	MPI-ESM1-2-HR	100km	r1i1p1f1	Müller et al. (2018)	https://doi.org/10.1029/2017MS001217
MPI-M	MPI-ESM1-2-LR	250km	r1i1p1f1	Mauritsen et al. (2019)	https://doi.org/10.1029/2018MS001400
MRI	MRI-ESM2-0	100km	r1i1p1f1	Yukimoto et al. (2019)	https://doi.org/10.2151/jmsj.2019-051
NUIST	NESM3	250km	r1i1p1f1	Cao et al. (2018)	https://doi.org/10.5194/gmd-11-2975-2018

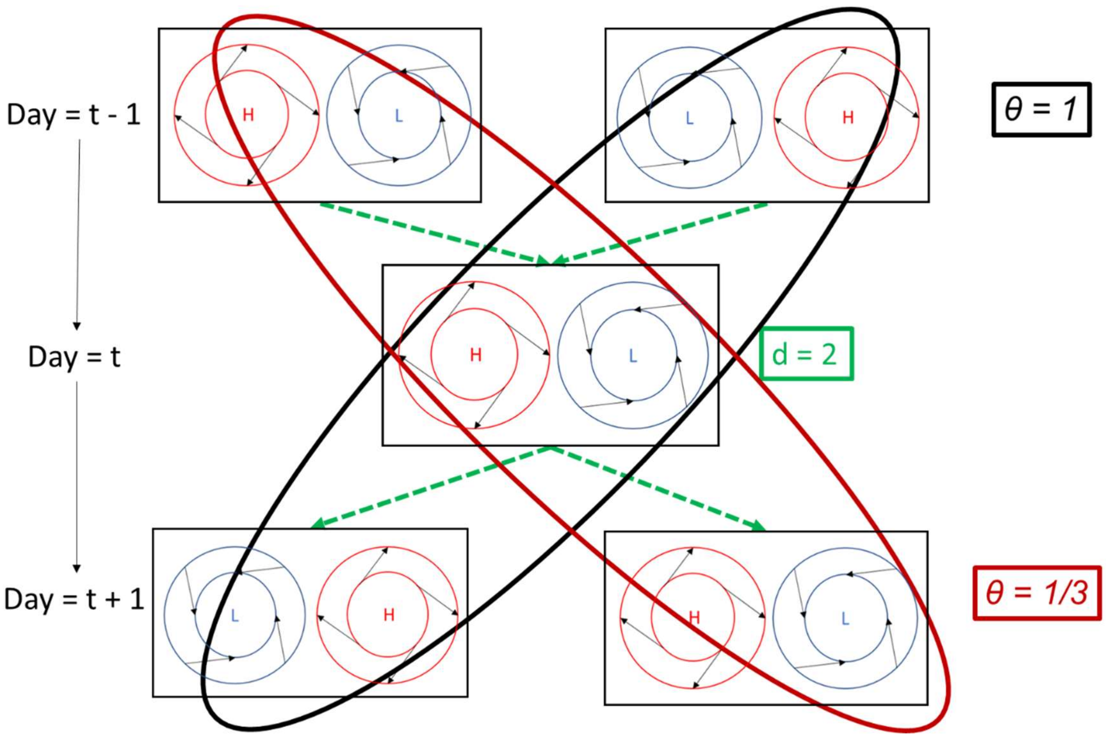

Figure S1 An intuitive schematic of the dynamical systems metrics computed for Day = t on made-up atmospheric states. The red ellipse represents a persistent atmospheric state (θ = 1/3), and the black ellipse represents a non-persistent state (θ = 1). There are two options for the atmospheric state to change from and to; therefore, d = 2. The schematic is inspired by a figure from Hochman et al.[1] and Rodrigues et al.[2].

References

1. Hochman, A., De Luca, P., Komacek, TD. Greater Climate Sensitivity and Variability on TRAPPIST-1e than Earth. *The Astrophysical Journal* **938(2)**, 114 (2022). https://doi.org/10.3847/1538-4357/ac866f
2. Rodrigues , D. et al. Dynamical Properties of the North Atlantic Atmospheric Circulation in the Past 150 Years in CMIP5 Models and the 20CRv2c Reanalysis. *Journal of Climate* **31(15)**, 6097-6111 (2018). https://doi.org/10.1175/JCLI-D-17-0176.1
